# Elicitor Application in Strawberry Results in Long-Term Increase of Plant Resilience Without Yield Loss

**DOI:** 10.3389/fpls.2021.695908

**Published:** 2021-07-01

**Authors:** Sanae Mouden, Johanna A. Bac-Molenaar, Iris F. Kappers, Ellen A. M. Beerling, Kirsten A. Leiss

**Affiliations:** ^1^Plant Health Team, Business Unit Greenhouse Horticulture, Plant Science Group, Wageningen University and Research, Wageningen, Netherlands; ^2^Laboratory of Plant Physiology, Plant Science Group, Wageningen University, Wageningen, Netherlands

**Keywords:** induced defense, metabolomics, thrips, methyl-jasmonate, greenhouse horticulture, strawberry cultivation, everbearer

## Abstract

For a first step integrating elicitor applications into the current IPM strategy increasing plant resilience against pests, we investigated repeated elicitor treatments in a strawberry everbearer nursery and cropping cycle under glass. During nursery methyl-jasmonate (MeJA), testing induction of defenses with plant bioassays was applied every 3 weeks. Thrips damage and reproduction by spider mites, whitefly and aphids were strongly reduced upon elicitor treatment. Subsequently, we applied MeJA every 3 weeks or based on scouting pests during a whole cropping cycle. Thrips leaf bioassays and LC-MS leaf metabolomics were applied to investigate the induction of defenses. Leaf damage by thrips was lower for both MeJA application schemes compared to the control except for the last weeks. While elicitor treatments after scouting also reduced damage, its effect did not last. Thrips damage decreased from vegetative to mature plants during the cropping cycle. At the end of the nursery phase, plants in the elicitor treatment were smaller. Surprisingly, growth during production was not affected by MeJA application, as were fruit yield and quality. LC-MS leaf metabolomics showed strong induction of vegetative plants decreasing during the maturation of plants toward the end of cultivation. Concurrently, no increase in the JA-inducible marker PPO was observed when measured toward the end of cultivation. Mostly flavonoid and phenolic glycosides known as plant defense compounds were induced upon MeJA application. While induced defense decreased with the maturation of plants, constitutive defense increased as measured in the leaf metabolome of control plants. Our data propose that young, relatively small plant stages lack constitutive defense necessitating an active JA defense response. As plants, mature constitutive defense metabolites seem to accumulate, providing a higher level of basal resistance. Our results have important implications for but are not limited to strawberry cultivation. We demonstrated that repeated elicitor application could be deployed as part of an integrated approach for sustainable crop protection by vertical integration with other management tactics and horizontal integration to control multiple pests concurrently. This approach forms a promising potential for long-term crop protection in greenhouses.

## Introduction

Strawberry production has globally increased over the last 10 years, both in acreage and yield per hectare [Mezzetti et al., 2018; FAOstat (http://www.fao.org/faostat/en/#search/strawberries)]. However, cultivation is under pressure in nurseries and fruit production due to stricter regulations increasingly restraining pesticide use, demanding new ways of pest and disease control. Integrated pest management (IPM) using different pest management approaches compatible with each other is adamant. A promising way in this regard is developing resistant cultivars (Mangandi et al., 2017). However, this remains a challenge in a polyploid crop like strawberry and requires a substantial time investment of at least several years (Mezzetti et al., 2018). Instead, using the natural potential of plants to defend themselves appears a promising way forward. By inducing their defense, plants become more resilient, making it more difficult for pests and diseases to establish and develop in the crop.

In Northern Europe, strawberries are increasingly cultivated under glass, allowing year-round, high-quality fruits (Mezzetti et al., 2018). Besides, the production of strawberries in tabletops protected with rain covers is quite popular. Strawberry nurseries are mostly outside still, but the most innovative frontrunner-growers are starting to experiment with protected plant nurseries under glass. Protected cultivation indoors or on covered tabletops is greatly advantageous for pest control. While keeping out pests and diseases allows for the introduction of natural enemies, both preventive and curative. The use of biological control in greenhouses is quite successful in practice but still has some challenges, particularly in the cultivation of strawberries requiring a relatively low-temperature regime compared to other greenhouse crops. This, together with the low amount of natural light in winter, hampers the establishment of the population and the development of most natural enemies (Sampson and Kirk, 2016; Clymans et al., 2017; Vervoort et al., 2017; Sampson, 2018). On the other hand, relatively high temperatures in summer allow for the rapid development of pest populations in the outdoor tabletops so that natural enemies cannot always keep up. This is especially true for strawberry summer-bearers with a relatively short cropping cycle. Induction of plant defenses may overcome these problems supporting and maintaining an equilibrium between pests and natural enemies (Pappas et al., 2017).

The induction of plant defense is regulated through plant hormones (Pieterse et al., 2012). Jasmonic acid and ethylene are generally associated with activation of defenses against pests, necrotrophic pathogens, and nematodes (Turner et al., 2002; War et al., 2012; Okada et al., 2015), while salicylic acid is associated with the activation of defenses against biotrophic pathogens (Palmer et al., 2017). Jasmonic acid is an important regulator of plant defenses. However, an important hurdle is the growth–defense trade-off (Koo, 2018). Increased plant defense usually comes at a fitness cost to growth. Nevertheless, in a recent meta-analysis of different studies, it appeared that induction through herbivore infestation reduced plant growth, photosynthesis, and reproduction upon feeding various insect guilds, but this depended on the plant development stage (Garcia et al., 2021). Negative effects on plant growth and development were only reported in the vegetative plant stage compared with the reproductive one.

Exogenous application of the active form of jasmonic acid, methyl jasmonate (MeJA), is known to induce plant defenses, resulting in increased plant resilience against herbivorous insect pests (Yu et al., 2018). In strawberry fruits, such application, at pre- and postharvest, resulted in accelerated fruit ripening as well as changes in levels of primary metabolites such as sugars and secondary metabolites such as anthocyanins and polyphenols, leading to improved fruit quality and shelf life (Giné-Bordonaba and Terry, 2016; Saavedra et al., 2016; Han et al., 2019; Zuñiga et al., 2020). Strawberry fruits infected with *Botrytis cinerea* experienced an up-regulation of marker genes associated with the induction of defenses (Valenzuela-Riffo et al., 2020). Less is known about the metabolome of strawberry leaves. So far, only total phenol content, antioxidant capacity, and organic acids have been described for some cultivars (Giné-Bordonaba and Terry, 2016). Concerning plant defense, strawberry leaf phenols were associated with lower feeding rates of spider mites (Wang and Lin, 2000; Luczynski et al., 1990), whereby elevated levels of phenols were reported to be observed upon feeding of this pest on strawberry leaves (Golan et al., 2017). Induction of defenses holds great potential as part of integrated pest management (IPM) strategy by enhancing the natural ability of a plant to defend itself against pests and diseases, i.e., plant resilience. However, little is known about the practicality of modulating plant resilience by elicitors during the whole strawberry cultivation cycle.

Therefore, this study investigated the induction of plant defenses, using the elicitor MeJA, against pests during the entire cropping cycle of greenhouse cultivated strawberries in a practice-like setting. We primarily focused on western flower thrips (WFT, *Frankliniella occidentalis*), one of the major pests in strawberry cultivation worldwide (Rahman et al., 2010; Sampson and Kirk, 2013; Strzyzewski et al., 2021). Moreover, we evaluated the potential of MeJA to induce plant defenses against spider mite as another cell-feeder and against phloem-feeding whiteflies and aphids. In general, direct thrips damage on leaves can potentially cause loss of photosynthetic capacity and crop yield in heavy infestations and indirect damage of virus transmission (Reitz et al., 2020). Specifically, in strawberries, thrips cause leaf and flower damage and bronzing and russeting of strawberry fruits leading to diminished shelf-life and fruit appearance (Rahman et al., 2010; Sampson and Kirk, 2013). Furthermore, the potential to modulate plant defenses by inducing host plant resistance to thrips has been stressed by Mouden and Leiss (2021). Therefore, to avoid thrips damage, it is important to inhibit thrips population build-up early in the vegetative leaf stages. We, therefore, concentrated on induced leaf defense, particularly looking into the following questions:

1. Does the exogenous application of MeJA induce plant leaf defense in both vegetative plants during nursery and mature plants during the production phase?
2. If so, is the application of MeJA concerning increased pest risk, as recorded by regular scouting, sufficient to decrease thrips damage?
3. Does induced leaf defense cause trade-offs such as reduced plant biomass, diminished fruit yield, and quality?
4. Which leaf metabolites are related to induction of defense by MeJA?
5. Which leaf metabolites are related to constitutive defense?

## Materials and Methods

### Effect of MeJA Application on Plant Resilience in the Plant Nursery, Greenhouse Experiment 2019

#### Cultivation

Strawberry seeds from the cultivars Delizzimo, Rowena, and Elan (ABZ Seeds, Andijk, The Netherlands) were germinated and grown in rockwool plugs at Beekenkamp Plants (Maasdijk, The Netherlands) until the two-leaf stage. Then, plugs were transferred to a greenhouse compartment at the experimental site of Wageningen University in Bleiswijk on the 29th of March. Upon arrival, plugs were transferred into nutrient solution saturated rockwool blocks (4 × 4 × 4 cm). When blocks reached 50% of the fully saturated weight, blocks were saturated again with nutrient solution applied from the bottom (eb-flood system).

One week after the experiment, plants were sprayed preventatively against mildew with Serenade [*Bacillus amyloliquefaciens* (str. QST 713)].

The climate conditions, such as temperature and relative humidity, realized during nursery are described in Supplementary Data 1.

#### Plant Resilience Treatments and Measurement of Plant Development and Growth

Plants were randomly assigned to three blocks. Per cultivar, blocks contained an equal number of plants. For each cultivar in each block, half of the plants were treated with MeJA (Sigma-Aldrich; 1 mM in 1% EtOH), the other half, as control, was mock-treated with 1% EtOH without MeJA Solutions were sprayed onto the plants until small droplets were visible on all leaves. Spraying was applied in the 1st, 2nd, and 3rd weeks of the experiment. In the 4th week, part of the plants was used to determine growth parameters, like length of the longest leaf stem, fresh and dry weight of the canopy. Another part was used to perform leaf bioassays with thrips (*Frankliniella occidentalis*), and the remaining plants were transferred to cages to perform whole-plant bioassays with thrips, spider mites (*Tetranychus urticae*), greenhouse whiteflies (*Trialeurodes vaporariorum*), or Buckthorn aphids (*Aphis nasturtii*). Each cage contained eight MeJA-treated and eight control plants of the same cultivars randomly placed.

#### Bioassays

Thrips, spider mites, and whiteflies were taken from our standard rearing on chrysanthemums, cucumber, and tomatoes. Rearing has occurred in small greenhouse compartments. Additional lighting (son-T) was given on dark days, but the day length was not extended. Relative humidity was set at 70%. Compartments were heated when the temperature dropped below 18°C. Compartments did not have windows, so cooling was not possible. Rearing of aphids occurred on strawberry plants in cages placed in the compartment used for the strawberry nursery (climate conditions in Supplementary Data 1). For each rearing, host plants were refreshed regularly.

The whole plant bioassay with thrips was conducted with 10 adult thrips per plant released into each cage. After 1 week, thrips damage on the leaves was visually evaluated using a score from 0 to 5; with 0: no thrips damage, 1: small spot on one leaf, 2; several spots on one leaf, 3 several spots on several leaves, 4 large spots, several leaves; 5 severe damage, all leaves with thrips damage (Bac-Molenaar et al., 2019).

The whole plant bioassay with spider mites was performed, attaching a piece of cucumber leaf with seven adult female spider mites to the largest leaf of each strawberry plant. After 1 week, the number of eggs was counted on all leaves of the plant using a binocular microscope.

The whole plant bioassay with whiteflies was executed by releasing five adults per plant into the cage. No sex was determined for these individuals, but in our rearing, most whiteflies are female. After 3 weeks, the number of eggs and number of larvae/nymphs (all stages together) was determined per plant.

The whole plant bioassay with aphids was conducted, attaching a piece of a leaf containing 10 aphids (mix of life stages) to the largest leaf of each strawberry plant. After 2 weeks number of individuals (all life stages) was counted on each plant. The establishment of aphids was only successful on the cultivar Delizzimo.

For the detached thrips leaf bioassay, the protocol was adapted from the dual-choice assay (Leiss et al., 2009a). A leaflet of a fully developed leaf from the middle of the strawberry foliage was placed on 1% water-agar in a pot with its abaxial surface up. After adding 10 adult thrips, the pot was closed with a lid partly containing a fine-mesh to prevent condensation. Thrips feeding damage was visually assessed in mm^2^. Per treatment, 10 replicates were used.

### Repeated Application of MeJA During Cropping Cycle—Greenhouse Experiment 2019–2020

#### Cultivation

**Figure 1:**
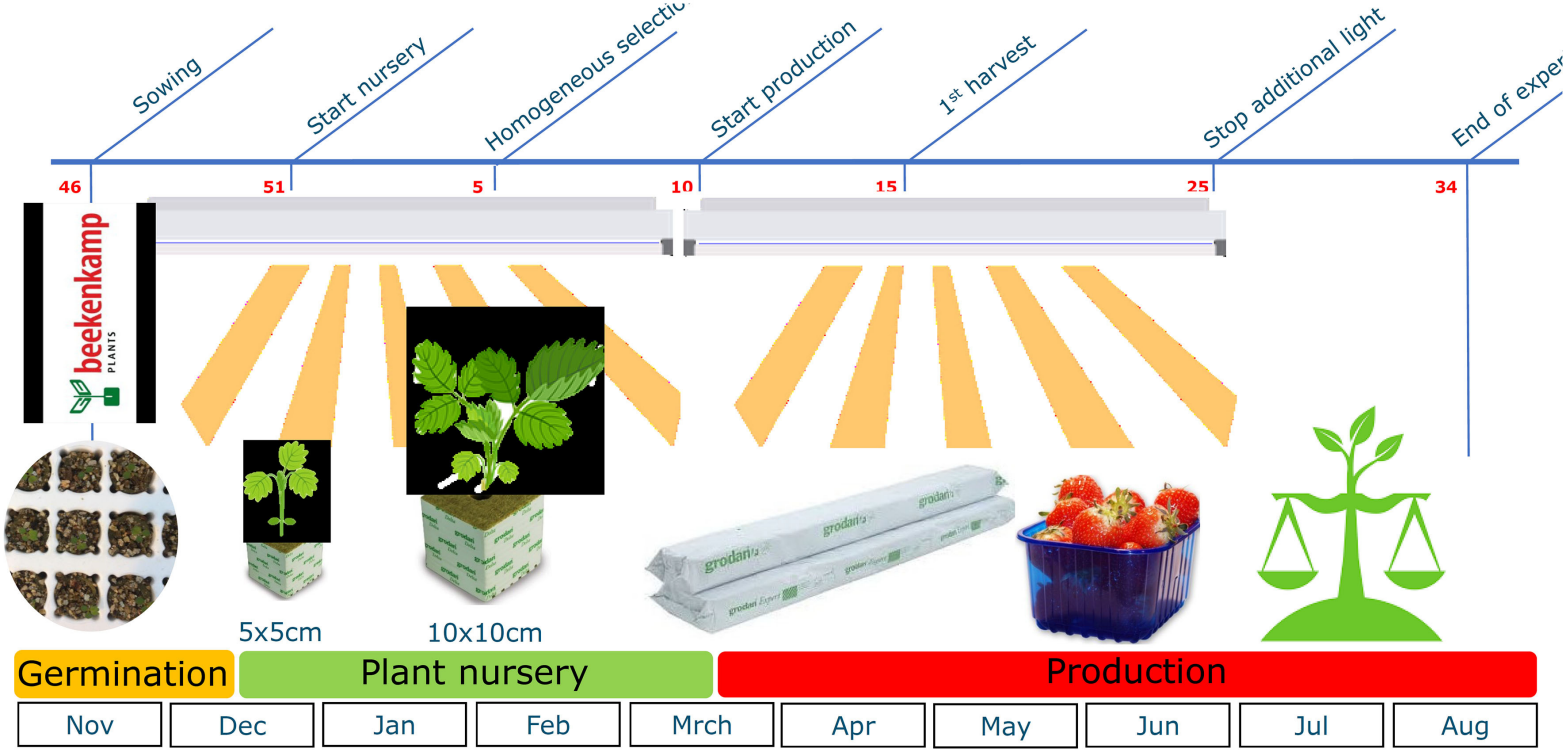
Strawberry cropping cycle 2019–2020.

Elicitor treatments to increase plant resilience of the everbearer strawberry cultivar Delizzimo, were evaluated during a whole cropping cycle (Figure 1) in 2019–2020. Although the dimensions of our trial compartment were relatively small in comparison to commercial greenhouses in the Netherlands, we aimed at achieving conditions as close as possible to real practice. Strawberry seeds were germinated on the 11th of November 2019 (week 46) as described above, transferred and transplanted in 5 × 5 × 5 cm rockwool blocks in week 51 with a density of 100 plants per m^2^. On the 30th of January 2020 (week 5), plants were transplanted in larger rockwool blocks (10 × 10 × 10 cm) with a density of 25 plants per m^2^. Until week nine, runners and trusses were removed regularly. On the 3rd of March 2020 (week 10), selected plants in comparable developmental stages were transferred to the production greenhouse. These five plants per running meter were placed, with a distance between two gutters of 1.20 m, resulting in a plant density of 4.5 plants per m^2^. Additional light (120 μmol/m^2^/s, Son-T) was provided until week 25 to obtain a day-length of 16 h and reduce differences in perceived light between the left and right sides of the compartment due to shadowing from the neighboring compartments. The climate conditions, such as temperature and relative humidity, realized during the cropping cycle are described in Supplementary Data 1.

A standing army of preventatively introduced natural enemies was chosen as a basis pest management strategy. Plants were scouted weekly to optimize pest management. A list of natural enemies that were released in the greenhouse is presented in Supplementary Data 2. In addition, we had to use Xentari (active compound, *Bacillus thuringiensis*, Bayer, Germany) once against caterpillars of the Turkish moth (*Chrysodeixis chalcites*). Also, diseases were scouted weekly. We applied several products to control powdery mildew. A list of products applied is provided in Supplementary Data 2.

#### Measuring Plant Development and Growth

In week 10, at the end of the nursery phase, 10 plants per block were sampled for destructive measurements. We determined the number of leaves for each plant, the number of trusses, length of the longest leaf stem, total leaf area using a leaf area meter, and plant dry weight after 36 h of drying at 50°C. The root index was established as a score of the amount of roots at the bottom of the rockwool on a scale from 0 to 5.

Light interception, as an approximation of photosynthesis and plant growth during the cultivation cycle, was measured three times each in weeks 18, 25, and 29. These measurements were performed on cloudy days, ensuring small variation in light intensity over time.

#### Measuring Fruit Production, Fruit Shelf Life, and Fruit Taste

The first fruits were harvested on the 6th of April (week 15). From then on, fruits were harvested twice a week until the 19th of August (week 34). Each harvested fruit was classified according to quality: Class I large: diameter >27 mm, good shape, no fungi. Class I small diameter <27 mm, good shape, no fungi. All other fruits which did not have any fungi and were of reasonable size were categorized as Class II. Fruits with fungi were discarded. In weeks 22, 27, 30, and 33, fruit shelf life was determined by storing Class I fruits for 4 days of 4°C and an additional day at room temperature. One box of fruits was stored for each block, resulting in 8 boxes per treatment. After storage, fruit quality was determined by scoring fruit damage and fruit rot in each box. In weeks 21, 24, 29, and 31 taste of the strawberries was determined as the two most important components for sweetness: soluble solids concentration (°Brix) and acid contents (titratable acidity) in the juice of strawberry fruits. In week 21, fruits of all blocks of the same treatment were pooled. In the other weeks, two pools were made for each treatment, each containing fruits from different blocks per treatment. A hand refractometer was used to determine the total soluble solids in each sample of blended pulp and is reported as the mean value of triplicate analyses. To determine the total titratable acidity levels, the potentiometric endpoint titration was applied. The blended pulp (5 g fresh weight) and distilled water (50 mL) were added to titrate with aqueous NaOH (0.1 M) to obtain pH 8.2. Total acid content was determined in mmol H_3_O^+^/100 g fresh weight.

#### Elicitor Treatments Inducing Plant Resilience

A completely random block design was used to study the effect of MeJA on plant resilience. Plant nursery was carried out on six tables. Each table was divided into three blocks, labeled “Control,” “Scouting,” and “MeJA.” In the production phase, each gutter (eight in total) was divided into three blocks, which were treated accordingly. Plants in each block were sprayed every 3 weeks (starting week 51 of 2019) as first observations pointed out that the effect of MeJA lasted about 3 weeks. “MeJA”-blocks were sprayed with 1 mM MeJA dissolved in 1% EtOH whereas plants in “Control”-blocks were sprayed with the corresponding mock solution of 1% EtOH. Blocks labeled as “Scouting” were sprayed with 1 mM MeJA upon scouting when pest pressure was increasing and not yet in balance with its natural enemies (Supplementary Data 1). This occurred in weeks 2, 5, 23, and 26. In the remaining weeks, plants in the “Scouting”-blocks were sprayed with the mock solution.

#### Bioassays

Starting in week 3, 1 week after each spraying, we performed a detached leaf bio-assay with thrips in the way described above with slight modifications: The leaflet was rinsed in MilliQ water and, upon drying, carefully checked for the presence of natural enemies and eggs of *Amblyseius limonicus* and *cucumeris* to avoid thrips predation. Subsequently, each leaflet was embedded in 1% water agar and infested with five adult thrips. For each treatment, 10 replicates were used. Thrips feeding damage was visually assessed in mm^2^ after 5 days. In weeks 19, 24, and 33, detached leaf bio-assays were also performed for *Botrytis cinerea*. The fungus was reared from a frozen stock culture of the strain BC 700 on a half-strength potato dextrose agar medium. For the bioassay, the same leaflet for the thrips bioassay was used, using 10 replicates per treatment. Two drops of 4 uL spore suspension containing 10^−6^ spores were placed at both sides of the main leaf vein ~2 cm apart from each other. The diameter of the developing lesions was determined after 6 d, averaging the diameters of both spots.

In weeks 9, 15, and 24, leaf material was sampled for metabolomics by flash-freezing it in liquid nitrogen and storage at −80°C. Since leaves of strawberry are composed of three leaflets, one leaflet each was used for the thrips and botrytis bioassays described above and one for metabolite analysis. In addition, a leaflet of an equivalent leaf was used for the analysis of polyphenol oxidase (PPO), a jasmonic acid defense-related marker protein (Mouden et al., 2020).

#### Polyphenol Oxidase (PPO)

Polyphenol oxidase (PPO) activity was determined toward the end of cultivation in week 24, as previously described by Mouden et al. (2020). One week after spray applications, 150 mg of fresh leaf material was sampled, flash-frozen in liquid nitrogen, and stored at −80°C until analysis. Leaf material was ground to a fine powder and extracted with 1.25 mL ice-cold potassium phosphate buffer (0.1 M, pH 6.8) containing 7% (w:v) polyvinyl polypyridine. To this homogenate, 0.4 mL of a 10% solution of Triton X-100 was added. Plant extracts were vortexed for 2 min and centrifuged at 11,000× *g* for 10 min at 4°C. The resulting supernatant was used directly as an enzyme source using chlorogenic acid as a substrate. The reaction mixture consisted of 5 μL enzyme extract and 1 mL of 2.92 mM chlorogenic acid dissolved in 0.1 M potassium phosphate buffer at pH 8.0. The rate of change of absorbance of this mixture was spectrophotometrically measured at 470 nm for 1 min (UV-1800 UV–VIS spectrophotometer, Shimadzu Europe GmbH, Duisburg, Germany). PPO activities were calculated from the linear slope and reported as changes in absorbance values per min per gram of fresh weight. Per treatment, 10 replicates were used.

### Statistical Analyses

Most data collected on plant growth, plant development, plant resilience, fruit quality, fruit shelve life, and fruit taste was ordinal or continuous, in most cases not normally distributed, even after transformation. Therefore, if one grouping factor was tested, we used a non-parametric Kruskal–Wallis test with subsequent pairwise comparisons by a Mann–Whitney *U* test. If two factors were tested, we performed generalized linear models (GLM) using Wald chi-squared tests with subsequent Mann–Whitney *U post hoc* tests. All statistical analyses were conducted with SPSS v. 26 software (IBM; SPSS Inc., Chicago, IL, United States).

### Metabolomics of Endogenous Semi-polar Metabolites

For untargeted analysis of semi-polar leaf metabolites, 20 mg of lyophilized leaf tissue was extracted with 500 μL 75% MeOH [0.125% formic acid (FA)] according to De Vos et al. (2007). After sonication and centrifugation, 10 μL aliquots were taken from each sample and combined for quality control (QC) sample analyzed multiple times throughout the randomized sample sequence.

Extracts were analyzed by accurate mass LC-MS as described previously by Jeon et al. (2021) with minor adaptations. An UltiMate 3000 U-HPLC system (Dionex, Sunnyvale, CA, USA) was used to create a 45 min linear gradient of 5–75% acetonitrile in a 0.1% FA water a flow rate of 0.19 mL min^−1^. Of each extract, 5 μL was injected, and compounds were separated on a Luna C18 column (2.0 × 150 mm, 3 μm; Phenomenex) at 40°C. A Q-Exactive plus-Orbitrap FTMS mass spectrometer (Thermo Fisher Scientific), operating at a resolution of 35.000 with scan-to-scan switching between negative (2.5 kV) and positive (3.5 kV) electrospray ionization (ESI) mode both over the m/z range 95–1,350, was used to detect eluting compounds. The QC sample was also analyzed in MSMS mode, in positive and negative mode, respectively.

Raw data files of –ESI mode LCMS analysis were subsequently processed in an untargeted manner using XCMS (https://xcmsonline/scripts.edu), including unbiased peak picking, alignment, and assembling of mass signals likely derived from the same metabolite. This unbiased processing resulted in putative compounds characterized by a specific in-source mass spectrum, including the putative molecular ion, isotope(s), and possible adducts and in-source fragments, at a specific retention time. All masses in the in-source spectra were searched for the presence of the calculated exact masses of the molecular ions [M–H]^−^ of all possible target compounds and their hexose and malonyl-hexose conjugates, allowing a mass deviation of 7 ppm. Annotation of selected compounds was performed manually by checking the relative intensities and accurate mass differences between m/z signals in the reconstructed full scan ESI-MS spectra. Processed mass signals were kept for further analysis when they were present in at least five out of six biological replicates per treatment.

#### Multivariate Analysis

We used six biological replicates for each treatment/time. Each biological replicate consisted of a pool of five plants. The preprocessed data were log-transformed, scaled and a one-way ANOVA per single metabolite was carried out using MetaboAnalyst 5.0 (https://www.metaboanalyst.ca/) determine which metabolites were different between conditions. Metabolites showing significance in the one-way ANOVA test were followed up by a Tukey's HSD *post hoc* test (alpha = 0.05) and used to perform principal component analysis (PCA) and hierarchical cluster analysis (HCA), reducing the dimensionality of the data to explore specific patterns of change in the metabolome as a result of plant developmental stage and MeJA applications. The HCA was performed using Pearson's correlation coefficient and Unweighted Pair Group Method with Arithmetic Mean (UPGMA). In addition, partial Least Squares Discriminant Analysis (PLS-DA) models were generated for each developmental stage to identify variable importance in projection (VIP) metabolites.

#### Metabolite Identification

Annotation of differentially regulated metabolites was performed based on matching the accurate masses of selected pseudomolecule ions to compound libraries, including Metlin (http://metlin.scripps.edu/), Metabolomics Japan (www.metabolomics.jp), the Dictionary of Natural Products (http://dnp.chemnetbase.com), and KNApSAcK (http://kanaya.naist.jp/KNApSAcK), using a maximum deviation of observed mass from the calculated mass of 15 ppm.

## Results

### MeJA Negatively Impacts the Performance of the Four Major Pests in Strawberry Cultivation

**Figure 2:**
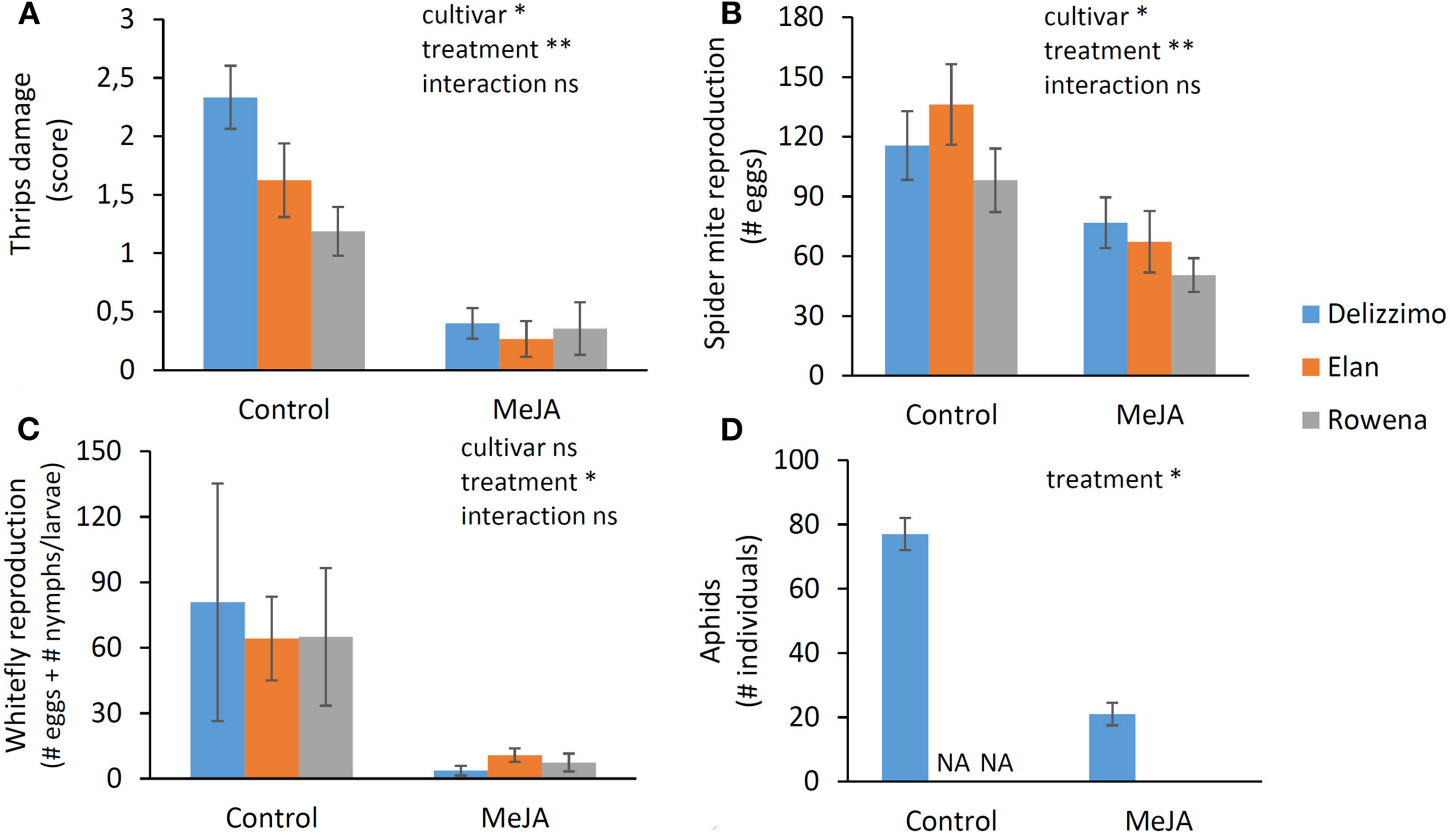
Effect of MeJA treatment on plant resilience against the four major pests during strawberry plant nursery as measured in whole-plant bioassays. **(A)** Thrips (*Frankliniella occidentalis*) leaf damage, scale score: 0 (*no damage*) to 5 (*severe damage*), **(B)** spider mite (*Tetranychus urticae*) reproduction (number of eggs per plant), **(C)** greenhouse whitefly (*Trialeurodes vaporariorum*) reproduction (number of eggs and number of larvae/nymphs per plant), **(D)** Buckthorn aphids (*Aphis nasturtii*) reproduction (number of individuals (all stages) per plant). Data present mean ± SEM (*n* = 8). Significant differences are indicated as *p ≤ 0.01, **p ≤ 0.001, based on GLM using a Wald chi-squared test.

The elicitor effect of MeJA on plant resilience against pests of different feeding guilds was evaluated in different strawberry cultivars in the nursery phase. While there was a significant cultivar effect (*p* ≤ 0.05) for thrips and spider mites, indicating that cultivars differed in their susceptibility to these pests, MeJA treatment affected all cultivars (*p* ≤ 0.01). Thrips and spider mites as cell feeders and greenhouse whiteflies and Buckthorn aphid as phloem feeders were negatively affected by MeJA treatment. In a whole-plant bioassay, thrips damage was 5 to 10 times lower in MeJA treated plants than the control plants (Figure 2A). The number of eggs laid by spider mites was 34–50% reduced in MeJA treated plants concerning the control (Figure 2B). Settlement of greenhouse whiteflies on MeJA treated plants was significantly lower (*p* ≤ 0.05) than the control, although the standard errors in the control treatment were relatively high (Figure 2C). Especially oviposition was strongly decreased. The aphid population on control plants showed a significantly stronger (*p* ≤ 0.05) 7-fold increase (from 10 to 77 individuals) in contrast to MeJA treated plants with a doubled population (from 10 to 21 individuals) (Figure 2D).

**Figure 3:**
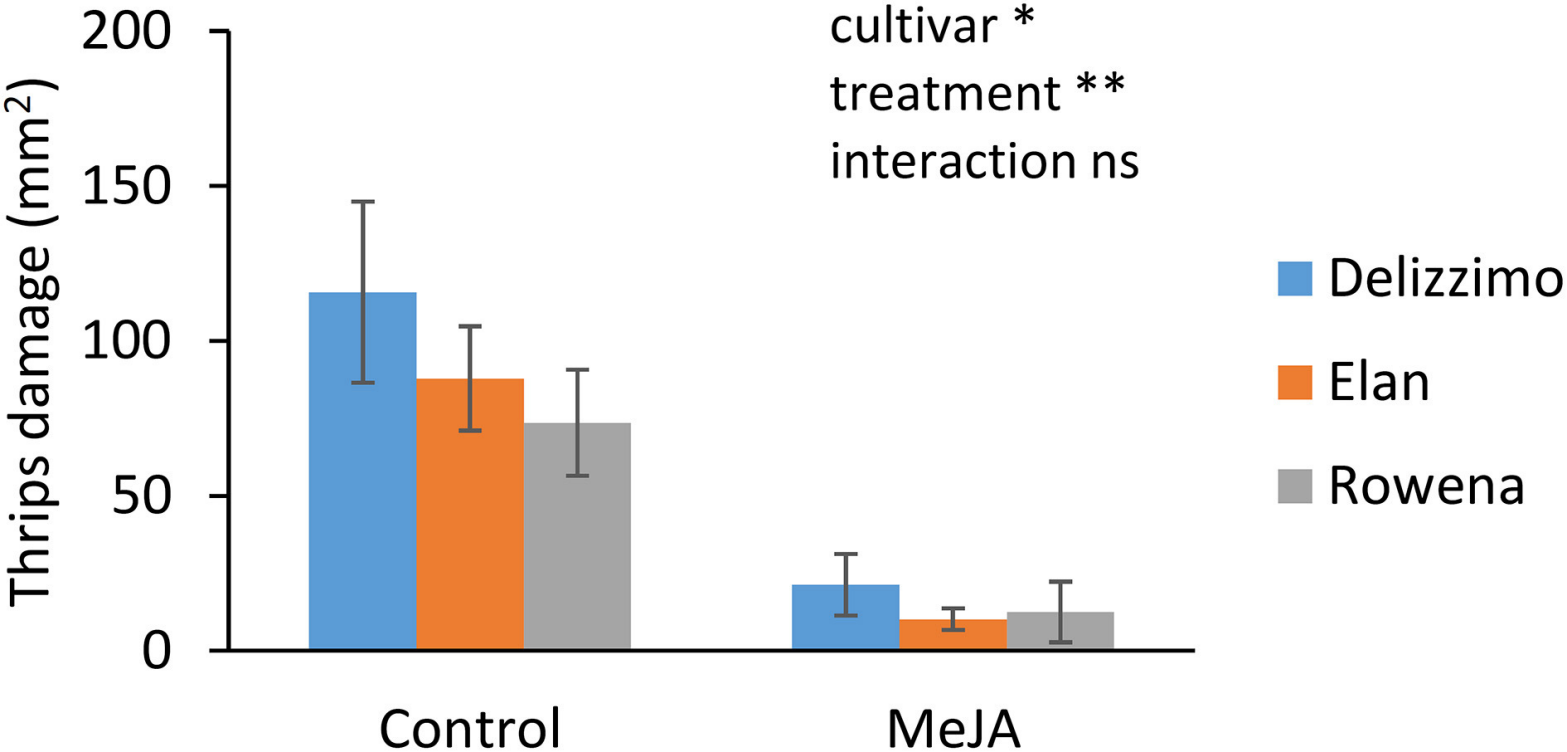
Effect of MeJA treatment on plant resilience at the end of a strawberry nursery measured by a thrips (*Frankliniella occidentalis*) detached leaf bioassay. Damage was visually estimated in mm^2^. Data present mean ± SEM (*n* = 10). Significant differences are indicated as *p ≤ 0.01, **p ≤ 0.001, based on GLM using a Wald chi-squared test.

We used a detached leaf bioassay to obtain data on plant resilience at the end of the nursery phase. In agreement with the whole plant assay, a significant effect (*p* ≤ 0.01) of MeJA treatment was observed for the three cultivars, leading to a more than 80% reduction of thrips damage compared to the control (Figure 3).

### Repeated Application of MeJA Results in Long Term Increase of Plant Resilience

**Figure 4:**
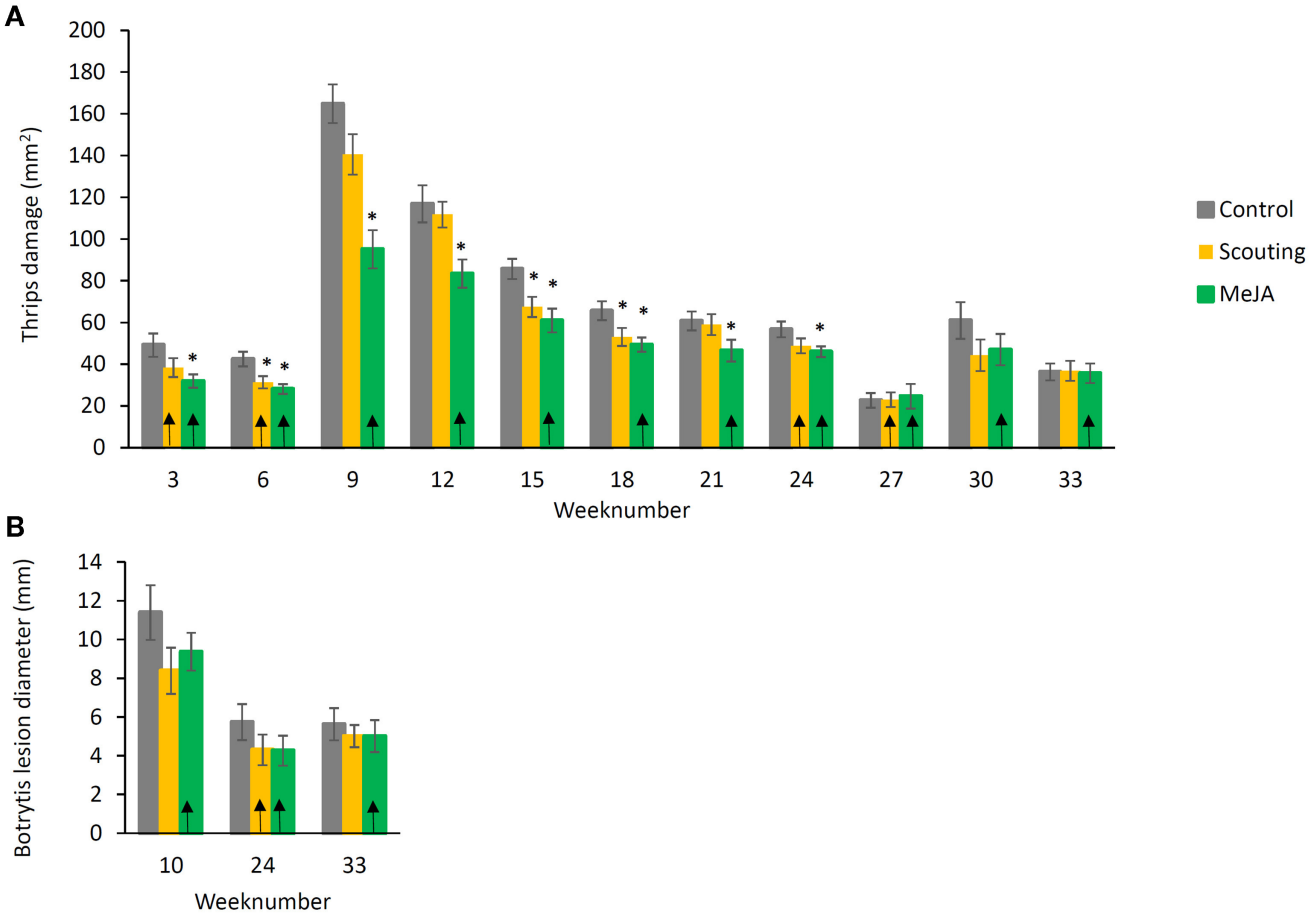
Plant resilience during the strawberry cropping cycle. **(A)** Thrips damage and **(B)** Botrytis lesions in detached leaf bioassays. Plant resilience against thrips was evaluated every 3 weeks. Plant resilience against Botrytis was evaluated in weeks 10, 24, and 33. Control: leaves from mock-treated plants, Scouting: leaves from plants treated with 1 mM MeJA when this was deemed necessary based on scouting, occurring in weeks 2, 5, 23, and 26. MeJA: leaves from plants that were treated with MeJA every 3 weeks. Upward arrows (↑) indicate that plants received MeJA treatment in the previous week. Data present mean ± SEM (*n* = 10). Significant differences are indicated as *p* ≤ 0.05 * based on a Kruskal–Wallis test.

Plant resilience was evaluated every 3 weeks during the cropping cycle using detached leaf bioassays. Significantly fewer thrips damage (*p* ≤ 0.05) was observed for the “MeJA” treatments compared to the “Control” for all bioassays except the last three (Figure 4A). Thrips damage strongly increased in week 9, whereafter it steadily declined toward the end of the cropping cycle. In treatment “Scouting,” MeJA was applied when this was deemed necessary based on pest pressure. Thrips damage on leaves from “Scouting” plants was significantly lower (*p* ≤ 0.05) than “Control” plants in weeks 6, 15, and 18. In weeks 9 and 12, the same trend was observed but was not significant. As a marker of JA plant defense responses, the polyphenol oxidase (PPO) activity was measured in week 24. One week before, plants had been treated with MeJA, and we observed a significant reduction in thrips feeding damage (*p* ≤ 0.05). However, this response was not accompanied by an increase in PPO levels (Supplementary Figure 1). Detached leaf bioassays with Botrytis did show less fungal growth on leaves of plants treated with “MeJA” or “Scouting,” but significance levels were not reached (Figure 4B).

### An Increase in Plant Resilience Does Influence Growth, but Not Production

**Figure 5:**
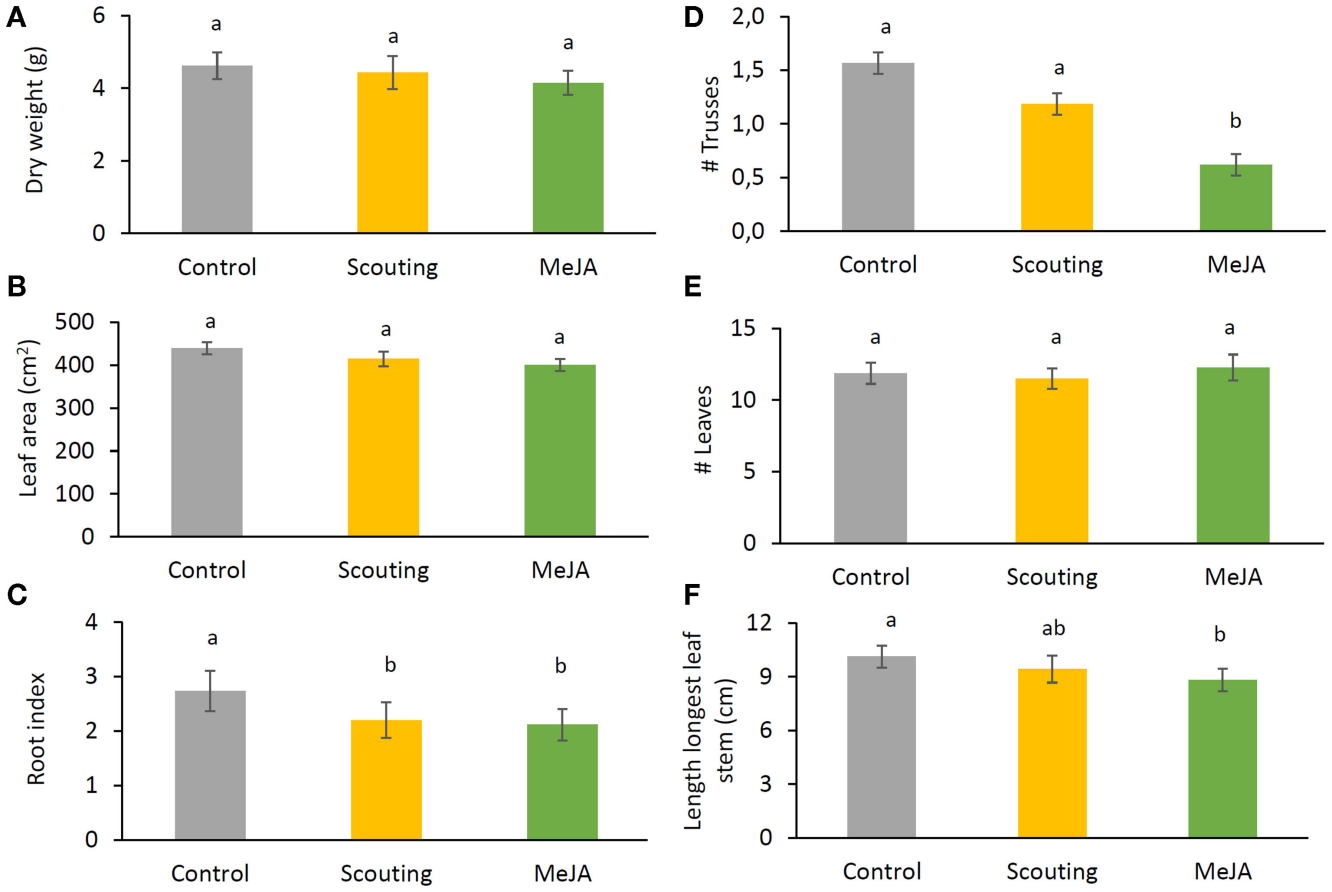
Effect of MeJA treatment on plant growth and development traits at the end of the nursery phase of the strawberry cropping cycle. **(A)** The dry weight of aboveground plant parts; **(B)** total leaf area; **(C)** root index. Score of the amount of roots at the bottom of the rockwool block on a scale from 0 to 5; **(D)** number of trusses; **(E)** number of leaves; **(F)** length of the stem of the longest leaf. Data present mean ± SEM (*n* = 10). Letters indicate significant differences between treatments at *p* ≤ 0.05 based on Kruskal–Wallis and subsequent pairwise comparisons using as Mann–Whitney *U* test.

**Figure 6:**
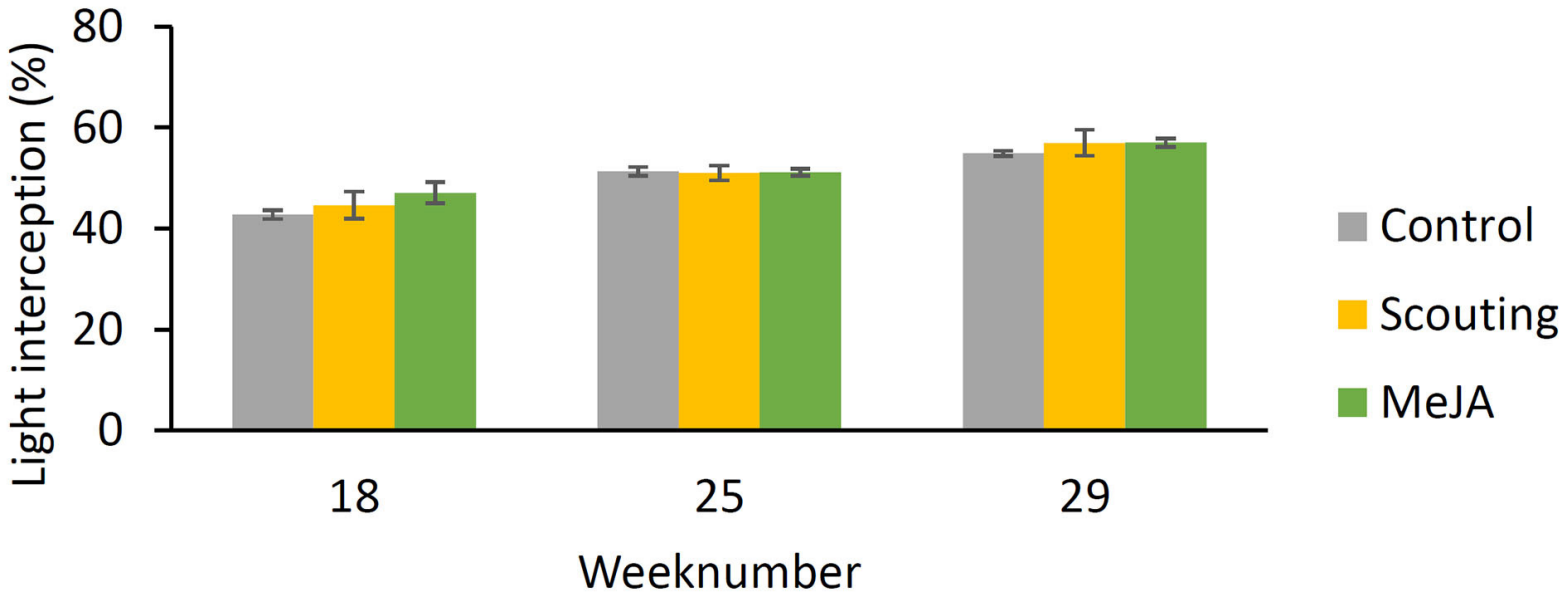
Light interception (%) during the strawberry production phase as determined in weeks 18, 25, and 29. Data present mean ± SEM (*n* = 3).

At the end of the nursery phase, plant growth and development were determined. No differences between treatments for aboveground dry-weight (Figure 5A) nor leaf area (Figure 5B) or number of leaves (Figure 5E) were observed. The length of the stem of the longest leaf was shorter (*p* ≤ 0.05), and the number of trusses was lower (*p* ≤ 0.05) for the MeJA treatment compared with the control (Figures 5D,F). The root index was lower (*p* ≤ 0.05) for plants in both treatments, “MeJA” and “Scouting” concerning control plants (Figure 5C). Thus, fewer roots grew through the rockwool block, reaching the bottom. Although light interception was increasing over time, no significant differences were observed between treatments (Figure 6).

**Figure 7:**
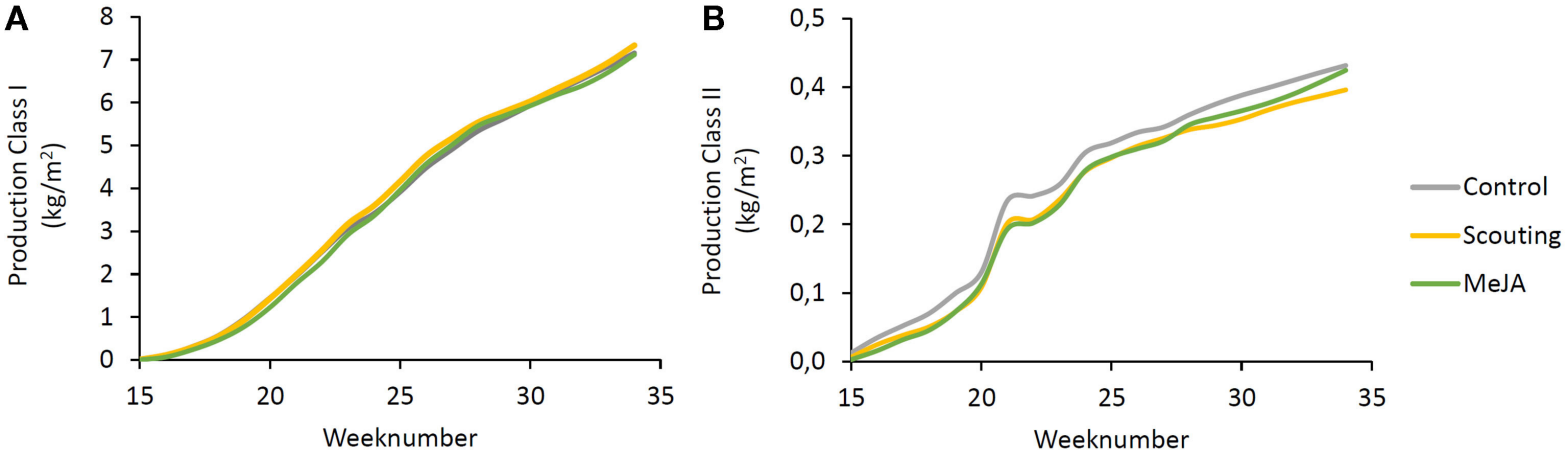
Strawberry fruit production over time. Cumulative weight of **(A)** first class and **(B)** second class fruits per week.

**Figure 8:**
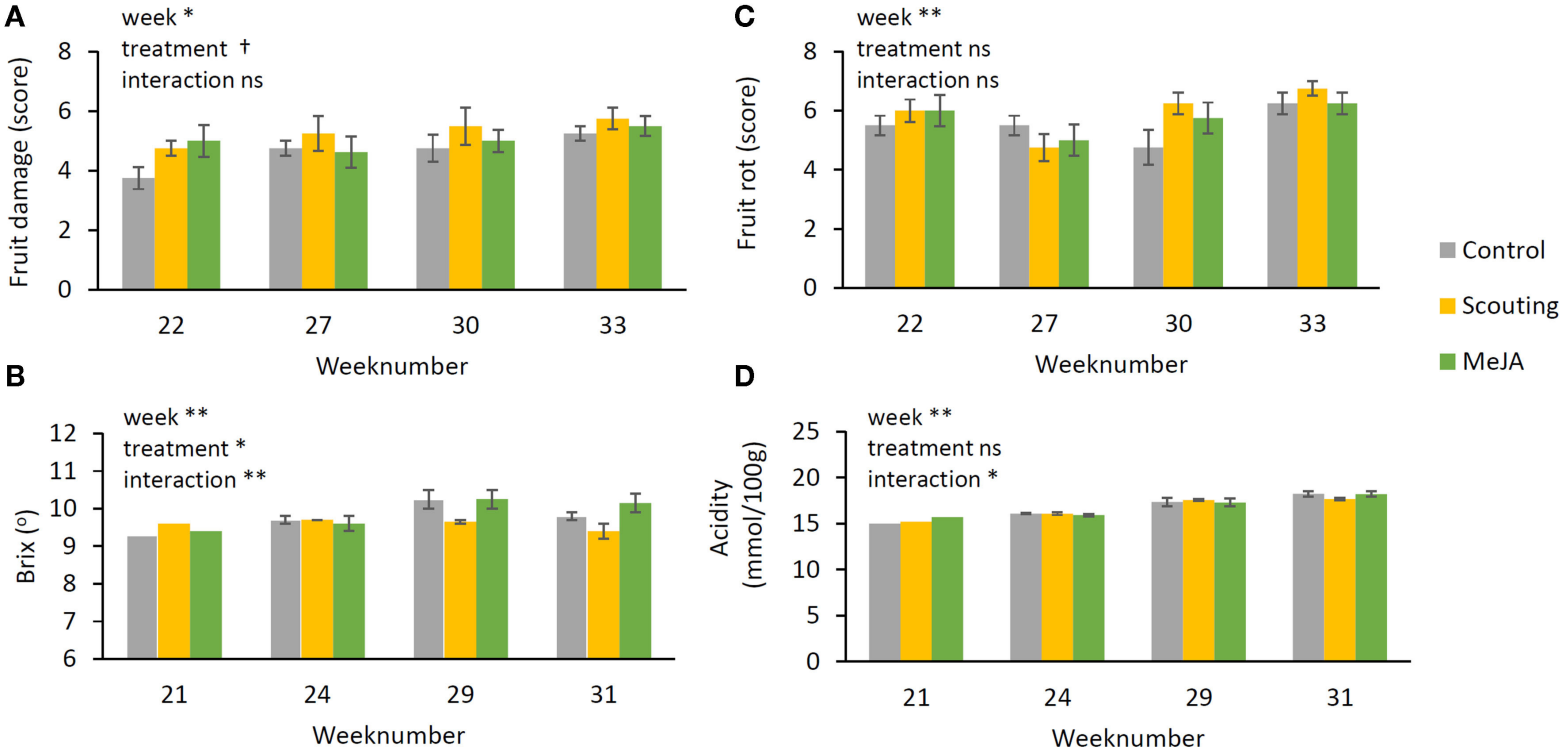
Shelf lives and tastes of harvested strawberry fruits during the production period. Shelf life was scored **(A)** fruit damage and **(B)** fruit rot. Scores are three: not acceptable, five: acceptable, seven: good. Data present mean ± SEM (*n* = 8). Taste was evaluated by determining the two most important components for sweetness **(C)** Brix (°) and **(D)** acidity (titratable acid in mmol/100 g fresh weight). Data present mean ± SEM (taste *n* = 1 for week 21 (mix of fruits of all eight blocks per treatment), *n* = 2 for weeks 24, 29, 31 (mix of fruits of 4 blocks per treatment). Significant differences are indicated as *p ≤ 0.05, **p ≤ 0.01, based on GLM using a Wald chi-squared test.

The weekly production of fruits did not differ between treatments. Also, the numbers of first and second-class fruits and their weight were not different between treatments (Figures 7A,B). Fruit damage (*p* < 0.05) was increasing over time (Figure 8A), and fruit rot was lower in weeks 27 and week 30 compared to week 33 (Figure 8B). While no differences between treatments were observed concerning fruit rot, fruit damage was significantly increased (*p* ≤ 0.05) in the scouting treatment, but the control did not differ from MeJA treatment. Brix and acidity, objective determinants of fruit sweetness, increased over time (*p* ≤ 0.01) (Figures 8C,D). Acidity did not differ between treatments (Figure 8D), but fruits of the “Scouting” treatment showed lower Brix levels than the other treatments (Figure 8C). However, the Brix of MeJA treated fruits did not differ from control fruits. These observations indicate that repeated application of MeJA did not affect fruit production, fruit quality, or fruit taste.

### Leaf Metabolome Is Changed During Plant Development

To obtain an overall view of adaptations of the leaf metabolomes upon MeJA treatment, we choose to use an unbiased LC-MS approach being inherently more sensitive above NMR, as we expected that changes in specific metabolite abundances might be small. LC-MS data were processed for negative and positive ionization mode separately, resulting in 1,627 and 378 mass signals. Technical reproducibility was high as Quality Control (QC) samples showed low variation. As biological variation within replicates was smaller than between experimental groups, we considered six replicates sufficient to capture a proper view of the metabolomes (Supplementary Figure 2).

**Figure 9:**
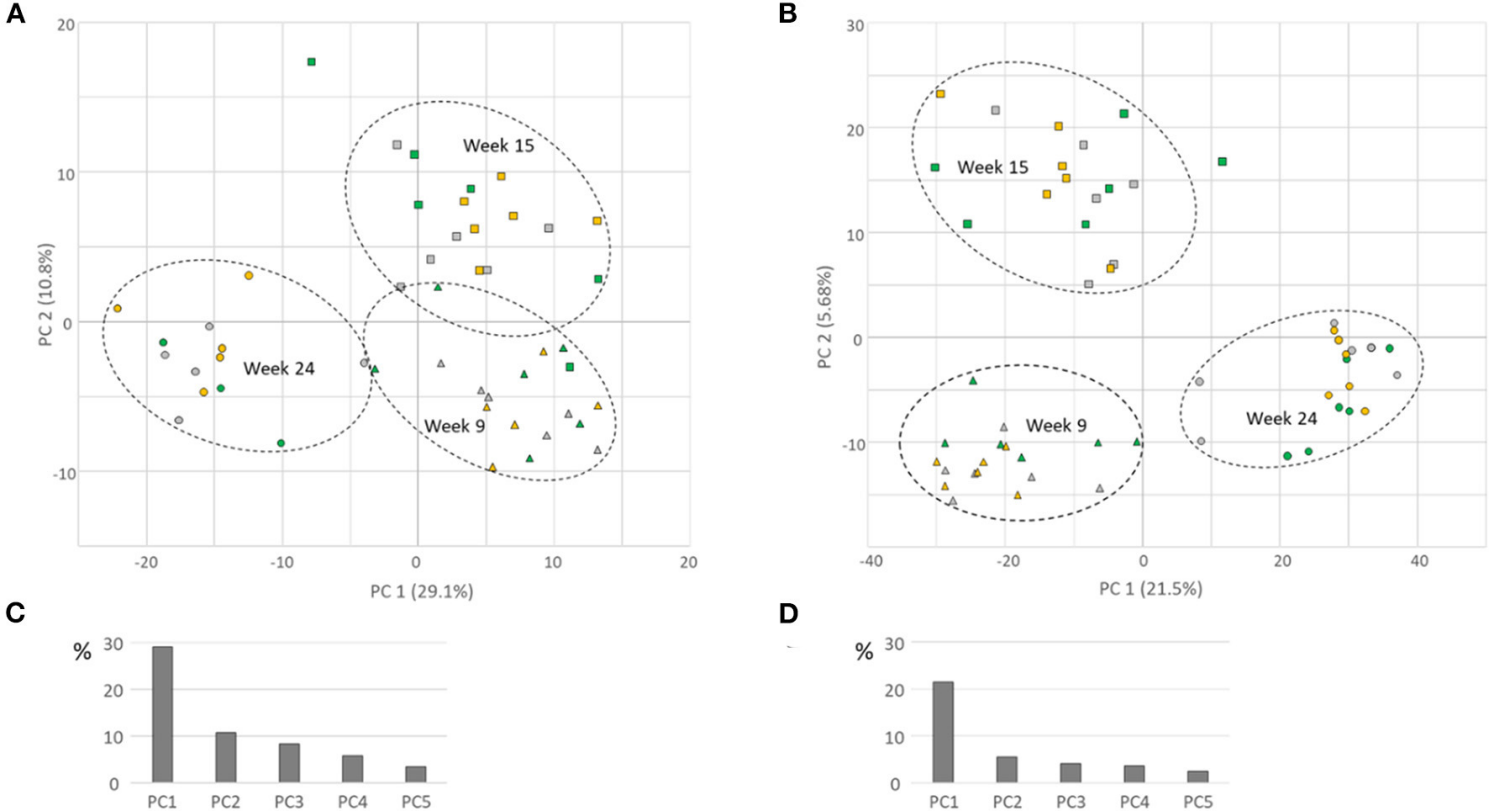
Leaf metabolomes of strawberry plants in week 9 (triangles), 15 (squares), and 24 (circles). Principal Component Analysis (PCA) of mass signals obtained in positive ionization mode **(A)** and negative ionization mode **(B)**. Bar diagrams show the variance that can be explained by the first five components (PCs) for positive **(C)** and negative **(D)** ionization mode. Gray symbols: mock-treated control plants; green symbols: MeJA-treated every 3 weeks; yellow symbols: MeJA treatment upon scouting.

Leaf metabolomes of strawberry plants differed during the developmental stage as visualized by unbiased PCA based on all detected mass signals normalized to the sum and log_2_ transformed (Figure 9). About 30–40% of the total variation in the dataset could be explained by the first two PCs for negative and positive ionization mode, respectively, separating leaf metabolomes from 9-week-old and 24-week-old plants among the first PC, while the second PC was additionally distinguishing 9-week-old plants from 15-week-old ones.

### MeJA Induced Impact on the Strawberry Leaf Metabolome Is Associated With Changes in Phenolics and Flavonoids Content

Considering the developmental variations in the leaf metabolomes of strawberry plants, we investigated the effect of MeJA applications on leaf metabolomes in weeks 9, 15, and 24 separately. The data were subjected to ANOVA with correction for multiple testing (Benjamini–Hochberg), and metabolites that were significantly different (*P* < 0.05 and fold change >2) between at least two treatments within each developmental stage were used to generate partial least squares discriminant analysis (PLS-DA) models (Table 1).

**Table 1:** Number of mass features included in the PLS-DA models and the qualitative descriptors of the PLS-DA models in Figure 10.

	**# features [*P* < 0.05; >2-fold]**	**ANOVA (% of total features)**	**# components for max accuracy**	**R^**2**^**	**Q^**2**^**
Week 9	1683	258 (15.33%)	5	0.99	0.89
Week 15	897	5 (0.56%)	2	0.98	0.73
Week 24	1200	13 (1.08%)	4	0.98	0.81

**Figure 10:**
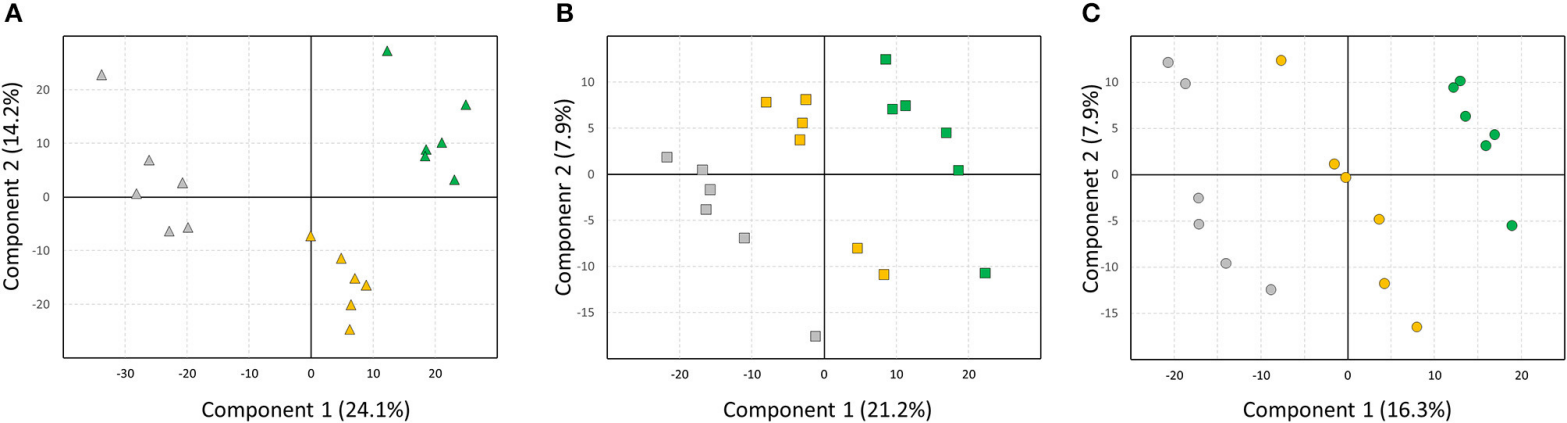
Partial least squares discriminant analysis (PLS-DA) based on methyl jasmonate-mediated changes in leaf secondary metabolites in strawberry plants in different developmental stages. **(A)** 9-week-old leaf metabolomes; **(B)** 15-weeks old; **(C)** 24-weeks. Gray symbols: mock-treated control plants; green symbols: MeJA-treated every 3 weeks; yellow symbols: MeJA treatment upon scouting. Cross-validation of the models: Q^2^ = 0.8, 0.75, and 0.75, respectively.

Of the 8,685 features detected by LC-MS, 3,780 (43.5%) were significantly different between at least two treatments in either developmental stage. A PLS-DA model of the selected metabolites present in 9-week-old plants demonstrated distinct clustering of the samples based on MeJA application, with latent variables 1 and 2 explaining 38.3% of the total variation (Figure 10A). Similarly, PLS-DA models for metabolomes of 15-week and 24-week-old plants indicated 29.1 and 24.2% of the variation, respectively, could be explained by the two dominant latent variables (Figures 10B,C).

**Figure 11:**
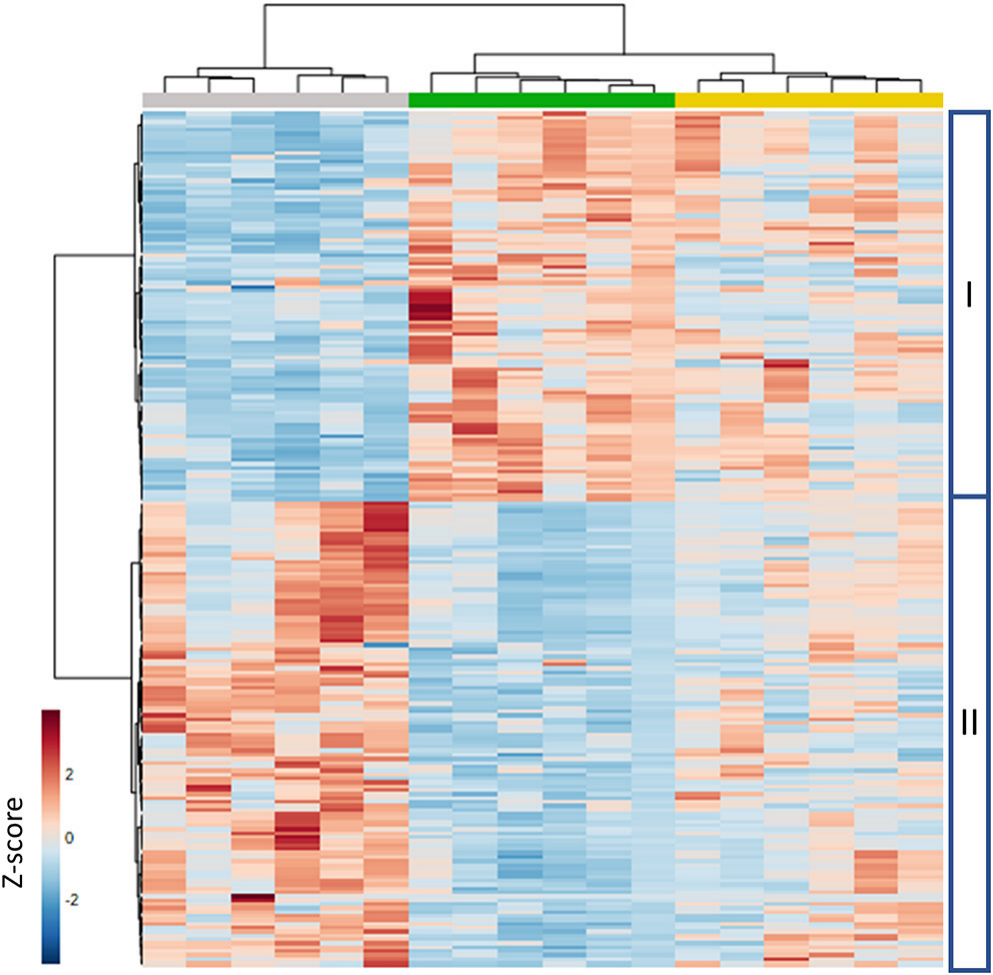
Hierarchical cluster analysis of selected (ANOVA, *P* < 0.05; fold change > 2) semi-polar metabolites in strawberry leaf metabolomes. A color-coded matrix represents the Z-score of the metabolite intensity in six biological replicates of strawberry leaves. I and II represent clusters of mass features induced (I) or reduced (II) upon MeJA application. Characteristics of the underlying metabolites are presented in Supplementary Table 2. Gray: mock-treated plants; green: MeJA-treated every 3 weeks; yellow: MeJA treatment upon scouting.

Next, we selected the features that contributed to the variables in projection for each of the PLS-DA models with a cut-off value >1.5 and used these to create a heatmap using Z-scores (Figure 11). Cluster features were associated with sample differences due to metabolites that were intrinsically more abundant in MeJA treated plants, while cluster II features were more abundant in mock-treated control plants. After removing adducts, tentative metabolite identification (Supplementary Table 1) showed that metabolites that were more abundant in MeJA treated plants than mock-treated control plants predominantly included glycosylated compounds derived from the phenylpropanoid pathway, including phenolic acids and flavonoid compounds such as flavanes, flavanones, isoflavones, and anthocyanidins. The most induced compound was a hepta-methoxy-flavanone ([M-H]^−^ 433.1508; MetLIN ID 53117) which was 111-fold more abundant in MeJA-treated leaves than control leaves. Also, catechin 3-O-rutinoside (flavan-3-ol) and pelargonidin 3-(6″-caffeylglucoside) ([M-H]^−^ 593.1305; MetLIN ID 46799, anthocyanidin) were significantly induced in MeJA-treated leaves compared to non-treated control leaves (Supplementary Table 1). Furthermore, multiple glycosylated phenolic acid compounds were more abundant in MeJA-treated leaves.

### Leaf Metabolites Related to Constitutive Defense Increase With Plant Maturity

**Figure 12:**
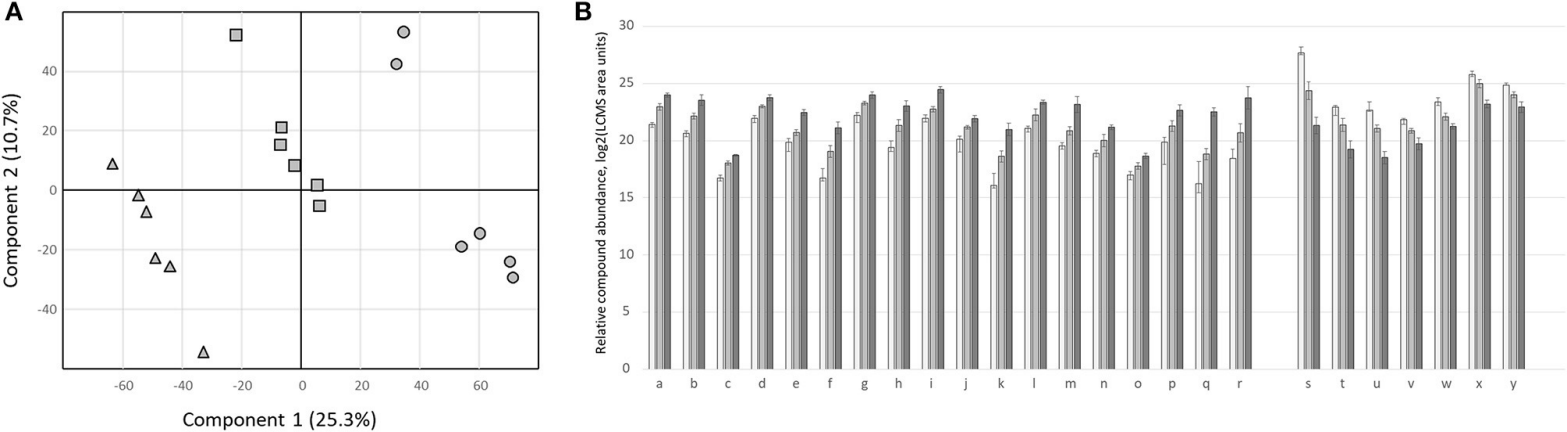
Leaf metabolome changes of control plants during development. **(A)** Partial least squares discriminant analysis (PLS-DA) based on secondary metabolites in control treatments of strawberry leaves at different developmental stages. Triangles 9-week-old leaf metabolomes; squares, 15-week-old; circles 24-week-old. Cross-validation of the model: Q^2^ = 0.9. **(B)** Relative abundance of metabolite features with highest variable in projection (VIP) score. White bars abundance in 9-week-old leaves; light-gray bars 15-week-old; dark-gray bars 24-week-old. Bars represent log_2_ values of means ± SD *N* = 6. Letters indicate different compounds, for details see Supplementary Table 3.

A PLS-DA model based on all detected metabolite features constitutively present in control plants of different ages demonstrated distinct clustering of the samples, and latent variables one and two explained 36% of the total variation (Figure 12A). Of the 25 metabolites that were most explanatory for the model (high variable in projection score), 18 showed increased abundance during development (Figure 12B). Metabolites relating to the developmental stage originate from different biochemical classes, including some flavonoid- and diterpene glycosides (Supplementary Table 2).

## Discussion

Modulation of plant defenses to increase host plant resistance becomes increasingly important within IPM (Mouden and Leiss, 2021). However, despite an extensive knowledge base, not much of existing basic research on natural plant defense strategies have been translated into applications that have been put into agricultural practice. To make the first step toward integrating elicitor application in current IPM practice, methyl jasmonate (MeJA) was either applied every 3 weeks or based on scouting of pests during the cultivation cycle of a strawberry everbearer. The present study is the first to report repeated pre-harvest foliar applications with the elicitor MeJA enhanced plant resilience against thrips and against the remaining major pests in strawberry: spider mite, whitefly, and aphids. Notably, we demonstrated that during production, these applications did not constrain fruit yield nor quality.

Artificial manipulation of Jasmonic acid (JA)-associated defenses has been reported to increase resistance against herbivores of multiple arthropod taxa and has been described extensively in several agri- and horticulturally important species (Dicke and Hilker, 2003; Miyazaki et al., 2014; Escobar-Bravo et al., 2017; Chen et al., 2018; Warabieda et al., 2020). While MeJA application has been described to induce resistance against the two-spotted spider mite (Warabieda et al., 2005), most studies concerning elicitor use in the Rosaceae family frequently evaluated postharvest effects of elicitor treatments rather than defense responses during cultivation. Congruent with the reported effects of JA-mediated plant-induced defenses on thrips in tomato (Chen et al., 2018, 2020) and chrysanthemum (Escobar-Bravo et al., 2017) showed that thrips feeding damage was significantly reduced upon repeated MeJA foliar sprays during the nursery phase. The three commercial cultivars varied inherently in thrips susceptibility, with the cultivar Delizzimo being more susceptible than the cultivars Elan or Rowena. Similar results were observed for spider mites where MeJA negatively affected reproduction. Moreover, MeJA sprays significantly reduced whitefly as well as aphid reproduction in comparison to control plants. Based on their feeding guild, spider mite and thrips are considered cell-content feeders whereas, aphids and whiteflies are phloem suckers. We thus demonstrated that both feeding types were negatively influenced by MeJA application in strawberries. While it is generally stated that insects activate the JA defense pathway, there are arguments that different insect feeding guilds elicit different plant hormonal signaling pathways, mediated by the phytohormones salicylic acid (SA) or JA. Chewing and cell-content feeding insects such as thrips predominantly induce JA-mediated defenses, whereas the SA-signaling pathway is induced by phloem-feeding insects (Pieterse et al., 2012; Lazebnik et al., 2014). The SA-pathway is considered to be the predominant signaling pathway during green peach aphid (*Myzus persicae*) infestation (Rodriguez et al., 2014), while JA-responsive genes were repressed (Kerchev et al., 2013). However, several authors suggest a role for both pathways in activating defenses against aphids (Cooper et al., 2004; Boughton et al., 2005; Selig et al., 2016). Similarly, the whitefly *Bemisia tabaci* has been shown to promote induction of SA-defenses while suppressing JA defense responses (Zarate et al., 2007; Walling, 2008; Estrada-Hernández et al., 2009; Puthoff et al., 2010). It is plausible that the antagonistic or synergistic function of the JA and SA defense pathways depends on the timing and level of the interaction between host plant and aphid species (Pieterse et al., 2009; Cao et al., 2014). Nonetheless, exogenously applied JA has been reported to decrease reproduction in whitefly (Shi et al., 2017; Esmaeily et al., 2020) and even virus transmission (Escobar-Bravo et al., 2016).

While JA treatment is known to induce defenses against necrotrophic fungi such as Botrytis in tomato (Bruce et al., 2017; Courbier et al., 2020), we did not observe any such effect in our leaf bioassays. However, although using lower concentrations compared to the ones we used, the effects described in the literature were obtained for 3-week-old vegetative plants and seedlings, respectively, while our first botrytis bioassay involved plants already 10 weeks old. Thus, MeJA application in older strawberry plants was not effective in inducing plant defenses against Botrytis. Young plants or tissues are frequently more responsive to defense elicitors than older plants or tissues (Cipollini and Redman, 1999; Köhler et al., 2015; Chen et al., 2018).

On the contrary, induction of plant defenses led to reduction of thrips damage of young plants in the nursery phase and older plants in the production phase, when MeJA was applied every 3 weeks. Also, incidental MeJA application after scouting reduced thrips feeding damage in the cultivation cycle. The positive effect, however, diminished over time when these plants were not repeatedly sprayed. In order to maintain durable protection, repeated MeJA applications during the cropping cycle are required. We observed a long-term increase of plant resilience from plant nursery until week 24 in the production. A long-term increase of plant resilience is particularly important in greenhouse horticulture, with usually relatively long cultivation cycles. Intriguingly, for an unknown reason, we observed a reduction in thrips feeding damage in non-treated scouting plants in weeks 15 and 18 while only plants from MeJA-plots were elicited. Moreover, we observed that thrips feeding symptoms were significantly higher in week 9 and decreased over time. From week 9 onwards first trusses became visible, indicating that the plant switched from the vegetative to the generative phase. In the generative phase, trusses, flowers, and fruits demand relatively many assimilates, possibly rendering plants more nutritious to thrips. Sugars may stimulate thrips feeding in plants (Scott Brown et al., 2002).

Strikingly, during the last 6 weeks of the production phase, no increase in plant resilience could be detected anymore. We assume that this may be the result of the age-dependent effects of the MeJA application. Induced plant responses have been reported to decline with age (Mao et al., 2017). Flower and fruit production over a longer period requires a substantial request for resources. Investment in growth comes at the expense of plant defense, as expressed by the growth-defense trade-off theory (Koo, 2018). At the same time, we showed that repeated elicitor applications resulted in shorter leaf length and less plant dry weight in the nursery. No reduction of plant growth nor fruit yield, or quality during production was observed. This is in line with the conclusions of a meta-analysis of induced defense costs by Garcia et al. (2021) stating that negative effects on growth occur during the vegetative but not during the reproductive plant stages. It is hypothesized that when the reproductive stage is reached, the plant prioritizes growth against the defense to guarantee a proper seed set. We did measure a decrease in defense metabolites induction from vegetative plants at the beginning to mature plants at the end of the cropping cycle. This is in line with our observation that polyphenol oxidase (PPO), an important JA-inducible marker, did not increase after MeJA application in week 24. Doan et al. (2004) also reported that PPO induction by foliar application of JA depended on plant developmental stage, indicating active defense of young tissues and potential shifts in defensive strategy during plant transition from growth to reproduction. However, we did not observe a concurrent increase in susceptibility to thrips. It has been proposed that in the early plant stages, when plants have relatively little biomass and potentially insufficient amounts of constitutive defense compounds, and active JA response is required for defense. As plants mature, constitutive defense metabolites seem to accumulate, providing a higher level of basal resistance (Barton and Koricheva, 2010; Mao et al., 2017). Indeed, the metabolome of the control plants embodying constitutive defenses present was significantly different between young and mature plants, as revealed by PLS-DA analysis. Changes were mainly dominated by increased abundance in flavonoids and diterpene glycosides, supporting a role in the reprogramming of constitutive leaf-based resistance in control plants over time. Indeed, diterpene glycosides (capsianosides) were negatively correlated with thrips preference and damage in an untargeted metabolomic profiling approach in Capsicum (Macel et al., 2019; van Haperen et al., 2021). Nevertheless, from week 25 onwards, we experienced caterpillar damage by the Turkish moth at the end of the cropping cycle—the only incidence during the whole cropping cycle we had to use insecticides. Concurrently, plants were infected with powdery mildew. It is generally stated that the two main plant defense pathways, JA and SA, work antagonistically (Pieterse et al., 2012). Thus, mildew infection in the MeJA treatment may be expected to be more severe. However, no differences in the percentage of infected fruits between treatments were observed (Supplementary Figure 3).

Exogenous application of MeJA has frequently been shown to alter the metabolome of plants by increasing the production of secondary metabolites, often in a defense-related context (Tugizimana et al., 2018). Partial Least Square-Discriminant analysis (PLS-DA) permitted to classify of leaf metabolomes based on MeJA treatment, exploring the effects per given timepoint. Heat map analysis of the differential metabolites revealed two clusters containing metabolites that were either induced or repressed upon foliar treatment with MeJA. Treatment with MeJA showed strong induction of metabolites compared to both scouting plants that were only incidentally treated with MeJA and control plants. Furthermore, multiple sugar-conjugated phenolic acid compounds were more abundant in MeJA-treated leaves, suggesting that treatment with MeJA shifted the glycosylation patterns of flavonoids. Many plant secondary metabolites in nature occur as glycosides (Bartnik and Facey, 2017). In general, glycosylation is known to affect the stability, storage, transport, availability, reactivity, and bioactivity of sugar acceptors. More importantly, glycosylation plays an important role in reducing phenylpropanoid toxicity, explaining the widespread occurrence in plant development and resistance/tolerance to major biotic and abiotic stresses (Bowles et al., 2005; Le Roy et al., 2016).

We tentatively identified 30 major potential metabolites induced upon MeJA application. Accordingly, main compound groups induced by MeJA were attributed to phenylpropanoid biosynthesis and included several flavonoid groups such as flavanes, flavanones, isoflavones, and anthocyanidins well as phenolic acids. Phenolic secondary metabolites are a major group directly involved in host plant resistance to insects and pathogens (Daayf et al., 2012; Mouden et al., 2017). In addition, they are involved in host plant resistance to thrips in different hosts such as carrot (Leiss et al., 2013) and tomato (Bac-Molenaar et al., 2019) as vegetables and chrysanthemum as an ornamental (Leiss et al., 2009b).

Among these metabolites, MeJA-triggered metabolic changes were largely characterized by a significant increase in levels of the annotated flavonoids, catechin 3-O-rutinoside, and pelargonidin 3-(6″-caffeylglucoside). The most abundant glycosylated forms in plants occur commonly as O-glycosides (Bartnik and Facey, 2017). The flavan-3-ol, catechin 3-O-rutinoside contains a rutinoside (6-O-α-L-rhamnosyl-D-glucosides) linkage bond via oxygen. Catechin itself is the major antioxidant compound in strawberry leaves (Buričová et al., 2011) and is considered among the major end products of the biosynthetic pathway in many plant species (Dixon et al., 2004). Catechins have been shown to play an important role in inducible defenses against foliar pathogen infection (Ullah et al., 2017; Wang et al., 2017) as well as herbivory (Kundu et al., 2018). In strawberry specifically, catechins are known to play a role in both constitutive (Yamamoto et al., 2000) and induced resistance to foliar pathogens (Feucht et al., 1992). Moreover, in buckwheat, catechins have also been reported to be induced upon JA treatment (Park et al., 2019). Pelargonidin 3-(6″-caffeylglucoside) belongs to a common class of organic anthocyanidins. Besides their role as natural pigments, glucoside derivatives which have putatively been implied as resistance related secondary metabolites (Karre et al., 2019). Another discriminatory marker with increased abundance in response to MeJA treatment was hepta-methoxy-flavanone. Although this poly methoxylated flavanone has not been previously linked to plant defenses, Roland et al. (2014) reported that six-methoxyflavanone acted as an antagonist of dietary bitter compounds.

## Conclusions

Integration of repeated application of methyl jasmonate in an IPM strategy, thus vertical integration of pest control measures, enabled increased plant resilience during a complete strawberry cropping cycle under glass, controlling thrips without reduction in fruit yield nor quality. In addition, reducing spider mites, whitefly and aphids indicates the potential of horizontal integration of elicitor application, controlling several different pests concurrently. While induced defense decreased with the maturation of plants during the cropping cycle, constitutive defense increased as measured in the leaf metabolome of control plants. Our data propose that when plants are relatively small in the early plant stages, a potential lack of constitutive defense compounds necessitates an active JA response for defense. As plants, mature constitutive defense metabolites seem to accumulate, providing a higher level of basal resistance. The results obtained have important implications for the cultivation of strawberry and other long-term cultivation crops in practice, showing that regular application of methyl jasmonate and potentially other elicitors during the crop cycle forms a very promising management tool for pest control a sustainable IPM strategy in the production greenhouses in general.

## Data Availability Statement

The datasets presented in this study can be found in online repositories. The names of the repository/repositories and accession number(s) can be found in the article/Supplementary Material.

## Author Contributions

JB-M, SM, EB, and KL: concept and design of experiments. JB-M and SM: execution of the experiments and analysis of data. IK: performance and analysis of metabolomics. JB-M, SM, IK, and KL: writing of the manuscript. All authors contributed to the article and approved the submitted version.

## Conflict of Interest

The authors declare that the research was conducted in the absence of any commercial or financial relationships that could be construed as a potential conflict of interest.
