# Supplementary material for: Elicitor Application in Strawberry Results in Long-Term Increase of Plant Resilience Without Yield Loss
Source: Front Plant Sci. 2021 Jul 1;12:695908. doi: 10.3389/fpls.2021.695908 (PMC8282209; doi:10.3389/fpls.2021.695908)
Supplement: Supplementary file 3 [file Data_Sheet_3.docx]

**
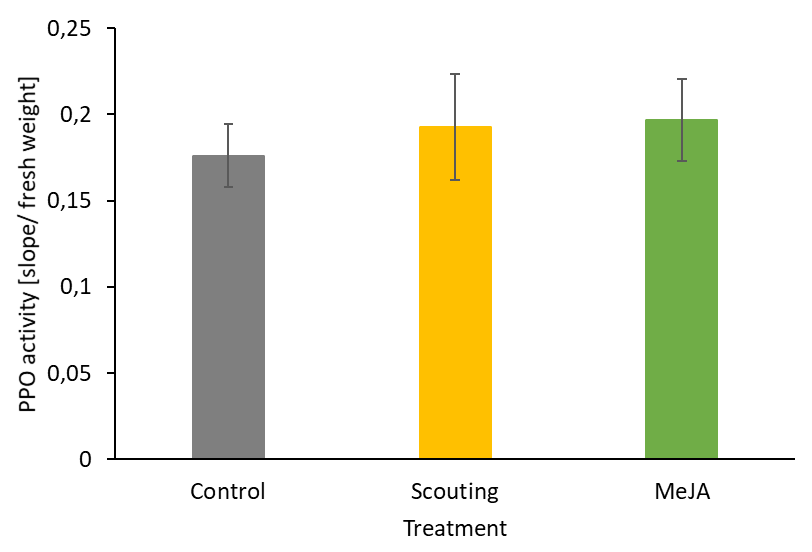
**

**Supplementary Figure S1.** Polyphenol oxidase activity (PPO) was measured one week after methyl jasmonate elicitation. Data are presented as mean ± SEM (n = 10). H(2) = 0.134, p = 0.935.


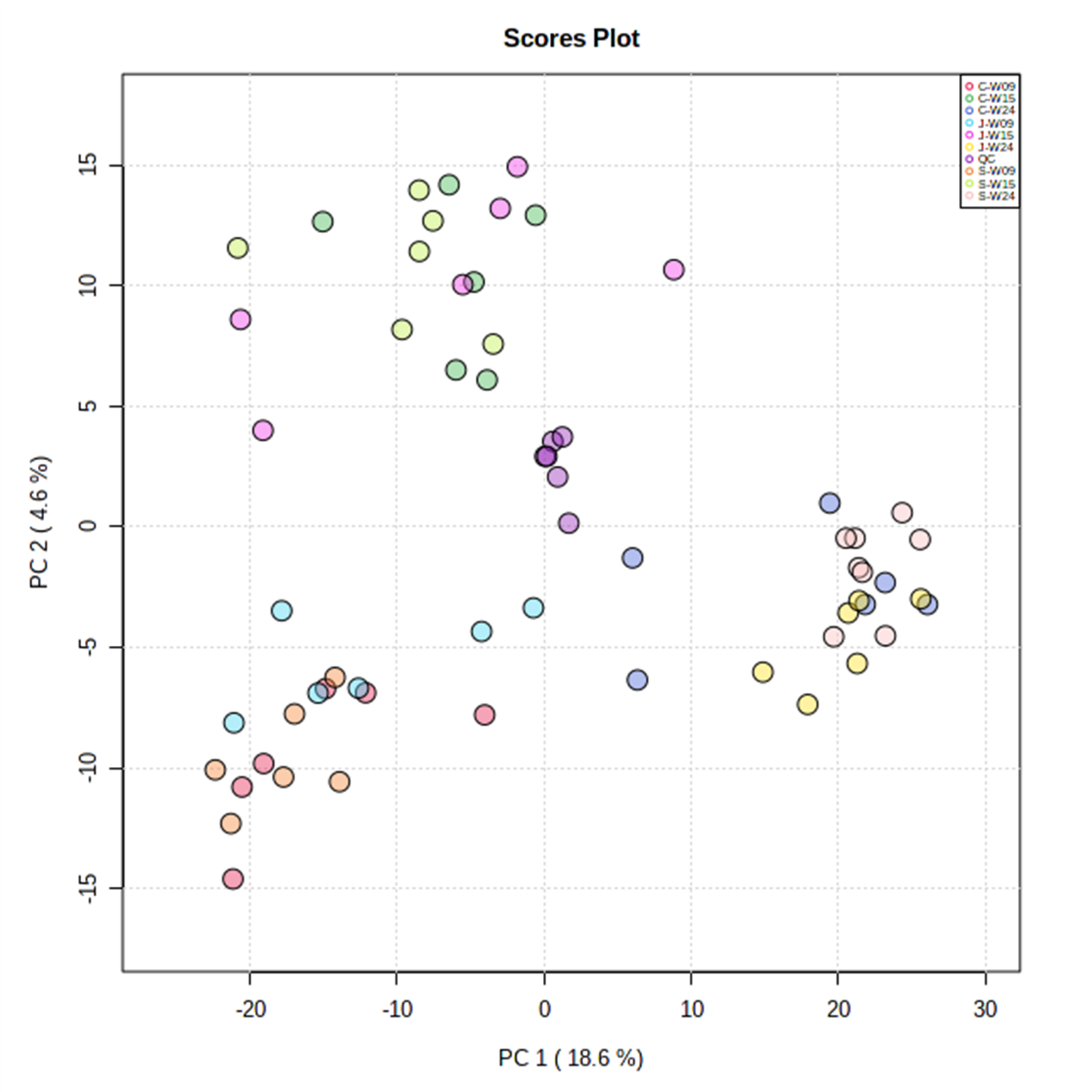


**Supplemental Figure S2** Principal Component Analysis (PCA) of strawberry leaf metabolomes from 9, 15 and 24-week-old plants. Score plot is based on features obtained from both negative and positive ionization mode. C: leaves from plants that mock-treated control plants, S: leaves from plants that were treated with MeJA when this was deemed necessary based on scouting. This was in week 2, 5, 23, and 26. J: leaves from plants that were treated with MeJA every 3 weeks. Similar aliquots of all samples combined form a Quality Control (QC) samples that was analyzed within the sequence, shows high technical reproducibility’s.


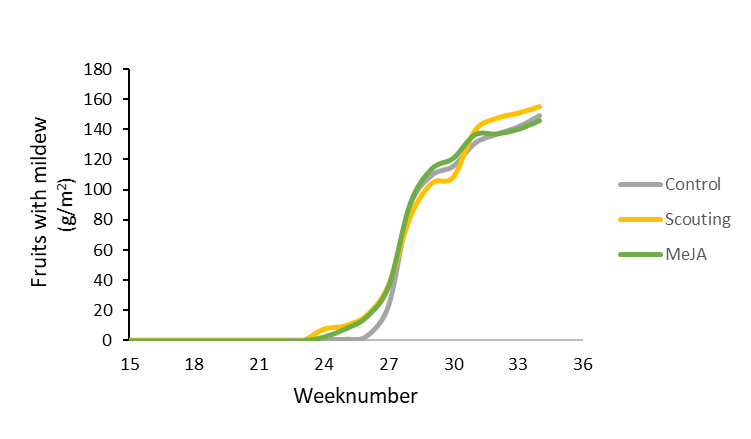


**Supplementary Figure S3.** Harvested fruits with mildew over time. Cumulative weight (g/m^2^) per week

**Supplemental Table S1** Metabolite features in strawberry leaf metabolomes most responsive to Methyl Jasmonate application (MeJA/Control) > 2-fold).

Mass Formula Putative Identification δ ppm Biochemical class log2(FC) MeJA/C

**Induced compounds**

434.158 C22H26O9 polymethylated flavanone 0 flavanones 6.80

478.058 C24H14O11 fucofuroeckol phenolic compound 6.72

529.545 unknown 6.09

594.138 C30H26O13 pelargonidin 3-(6''-caffeylglucoside) 0 anthocyanidins 5.69

526.222 C27H32N2O6 solifenacin isoquinoline alkaloids 4.24

366.096 C17H18O9 furocoumarinic acid glucoside 4 phenolic glucoisde 4.15

175.963 unknown 3.91

446.178 C20H30O11 crosatoside 0 phenolic O-glycoiside 3.68

344.038 C13H12O11 O-galloyl-galactarolactone 0 galloyl ester 3.34

336.132 C16H20N2O6 semilepidinoside 22 phenolic O-glycosides 3.19

408.142 C20H24O9 ginkgolide 0 diterpene 3.17

197.150 unknown 3.14

622.242 C34H38O11 phenolic O-glycoside 2 phenolic O-glycoside 3.10

524.190 C25H32O12 ligustroside 1 phenolic O-glycoside 3.03

598.204 C27H34O15 catechin 3-O-rutinoside 24 flavan-3-ol-glycoside 2.75

430.272 C26H38O5 persicaxanthin 0 diterpenoids 2.56

459.183 unknown 2.54

756.169 C39H32O16 quercetin 3-(3'',6''-di-p-coumarylglucoside) flavonoid glucoside 2.51

642.362 unknown 2.49

412.173 C20H28O9 diterpenoid 0 diterpenoids 2.43

458.179 C21H30O11 hydroxychavicol rhamnosyl-glucoside 1 phenolic glycoside 2.29

400.080 C20H16O9 tetrahydroxy-methylsuccinoyl flavone 0 flavone 2.26

590.294 C36H38N4O4 dimethylprotoporphyrin dimethyl ester 8 porphyrins 2.25

450.153 C22H26O10 auriculoside 0 flavan glucoside 2.25

344.090 C18H16O7 eupatorin 1 flavone 2.18

385.080 unknown 2.15

346.063 C17H14O8 tetrahydroxy-dimethoxyflavone flavone 2.15

647.120 unknown 2.14

520.180 C22H32O14 citrusin 1 lignan glycoside .09

196.146 C12H20O2 dodecadienoic acid 0 fatty acid derivative 2.09

556.246 C34H36O7 ingenol 3,20-dibenzoate 11 diterpene derivative 2.06

241.140 unknown 2.04

450.092 C20H22N2O6S2 exserohilone sulphur compound 2.02

714.408 C37H62O13 cyclopassifloside 15 triterpene saponin 2.02

534.210 C27H34O11 hydroxy-methoxy-prenylflavan -O-glycoside1 flavan-3-ol 1.77

602.127 C28H26O15 tetrahydroxyflavanone-galloylglucoside 2 flavanone 1.77

376.204 C25H28O3 ovaliflavanone 1 flavonone 1.69

unknown 1.48

300.063 C16H12O6 diosmetin 0 Isoflavone 1.37

346.069 C17H14O8 syringetin 2 O-methylated flavonol 1.31

405.076 C12H23NO10S2 glucocleomin 5 sulphur containing compound 1.28

241.106 C10H15N3O4 5-methyldeoxycytidine 12 dinucleotide 1.27

358.069 C18H14O8 polymethoxylated isoflavone 0 isoflavone 1.08

**Repressed compounds**

440.0504 unknown -7.39

308.0104 unknown -7.02

475.3197 unknown -6.75

288.0634 C15H12O6 dihydrokaempferol 1 flavonol -6.74

338.0313 unknown -4.57

934.1804 C44H38O23 sennoside 14 anthracenecarboxylic acid -3.36

531.136 unknown -2.92

446.1424 C19H26O12 lucuminic acid 0 o-glycosyl compound -2.66

439.2206 C22H33NO8 parsonsine 13 pyrrolizidine alkaloid -2.61

506.2727 C24H42O11 megastigmene-diol apiosyl-glucoside 0 saccharide fatty acyl glycoside -2.49

354.0587 C15H14O10 caffeoyl isocitrate 7 -2.47

448.2308 C21H36O10 linalool xylosyl-glucoside 1 terpenoid glycoside -2.45

470.1213 C24H22O10 pongamoside 1 furanoflavonoid glucoside -2.44

410.1002 C22H18O8 epicatechin 3-O-hydroxybenzoate 1 catechin -2.40

724.4398 C39H64O12 smilanippin 0 steroidal saponin -2.33

544.252 C26H40O12 cinncassiol glucoside 0 terpenoid glycoside -2.33

314.1002 C14H18O8 glucovanillin 1 phenolic glycoside -2.26

406.057 C15H18O11S O-coumaroyl glucose-O-sulfate 11 hydroxycinnamic acid glycoside -2.02

160.110 C8H16O3 hydroxy caprylic acid 0 fatty acid derivative -0.20

572.047 C22H20O16S isorhamnetin 3-glucuronide-7-sulfate 23 flavone -0.31

145.053 C9H7NO Isoquinoline N-oxide 0 isoquinoline alkaloid -0.52

290.131 C20H18O2 methyl-pentenyl anthraquinone 14 anthraquinone -0.92

**Supplemental Table S2.** Top-25 metabolites that most strongly differ in abundance during development of strawberry plants

**Compound ID [M-H]- d ppm putative identification**

a 288.0446 6 Isocarbophos C11H16NO4PS salicylate compound

b 319.1402 1 Valproic acid glucuronide C14H24O8 fatty acid derivative

c 636.3973 21 2'-O-Methylphaseollinisoflavan C21H22O4 Flavan

d 505.2074 1 Limonoate C26H34O10 dicarboxylic acid

e 522.1916 0 Gibberellin glucosyl ester C26H36O11 diterpenoid glycoside

f 271.0616 1 (-)-Naringenin C15H12O5 Flavanone

g 552.2173 unknown

h 619.2799 6 Asclepin C31H42O10

i 521.1878 0 Kanokoside  C21H32O12

j 506.2107 15 Bitolterol C28H31NO5

k 689.3394 0 diterpene glycoside C32H52O13 diterpenoid glycoside

l 479.2138 10 unknown

m 545.1885 11 Pilosanol  C28H30O10

n 480.2456 12 Pentazocine glucuronide C25H35NO7

o 380.2011 17 Symlandine C20H31NO6

p 281.1397 1 Hexenol O-glucopyranoside C12H22O6

q 526.238 24 unknown

r 635.2741 3 Calafatimine C38H40N2O7

s 131.0461 27 hydrocinnamic acid C9H10O2

t 426.034 unknown

u 324.0915 6 N-Glycolylneuraminic acid C11H19NO10

v 1085.0762 unknown

w 231.0875 2 perseitol C7H16O7

x 104.0352 1 serine C3H7NO3

y 542.5361 unknown
